# Two Cases of Ectopic Pregnancy Mimicking Gestational Trophoblastic Disease

**DOI:** 10.1155/2020/2417428

**Published:** 2020-06-16

**Authors:** Haruka Suzuki, Yoshitsugu Chigusa, Junzo Hamanishi, Masaki Mandai, Eiji Kondoh

**Affiliations:** Department of Gynecology and Obstetrics, Kyoto University, 54 Shogoin Kawahara-cho, Sakyo, Kyoto 606-8507, Japan

## Abstract

A well-known typical feature of ectopic pregnancy is an evident gestational sac structure outside of the uterus. However, some cases show atypical appearance that is described as a heterogeneous hypervascular mass. We report two cases of ectopic pregnancy that presented heterogeneous findings mimicking gestational trophoblastic diseases but were correctly diagnosed as ectopic pregnancies on MRI. The first case was an interstitial pregnancy in which the patient underwent surgical treatment. The second case was a cesarean scar pregnancy that was treated conservatively but showed spurious enlargement of pregnancy-related lesions after the treatment. Both cases lacked myometrial invasion on MRI, and the patients were diagnosed with ectopic pregnancies. Invasive findings on MRI may discriminate ectopic pregnancy from trophoblastic tumors and avoid unnecessary hysterectomy.

## 1. Introduction

Ectopic implantation occurs in approximately 1−2% of all pregnancies. Fallopian tube pregnancy accounts for 90% of ectopic pregnancies. Other unusual implantation sites include the uterine cervix, interstitium, ovary, abdominal cavity, and cesarean scar tissue [1]. Magnetic resonance imaging (MRI) is a useful diagnostic modality for detection of an ectopic pregnancy. Typically, an extrauterine gestational sac (GS) is exhibited as a high-intensity cystic structure surrounded by a thick wall on T2-weighted images and is enhanced in the early phase [2]. However, not all cases of ectopic pregnancy show these typical image findings.

We report two cases of ectopic pregnancy that presented heterogeneous findings mimicking gestational trophoblastic disease (GTD) on MRI.

## 2. Case 1

**Figure 1:**
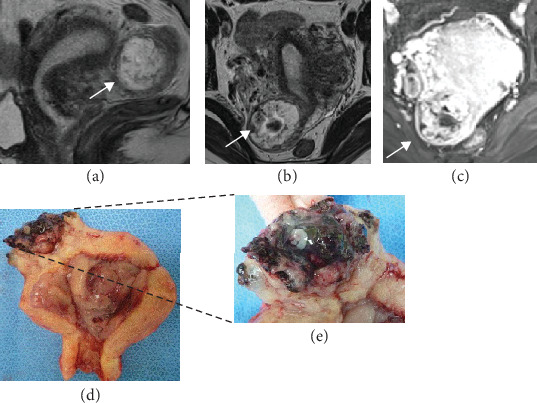
MR image performed at 7 weeks and 5 days in case 1 shows an interstitial pregnancy. A heterogeneously-enhanced hypervascular mass is identified on the right side of the uterine fundus. The mass measures 52 mm, which is larger than a GS at the corresponding estimated gestational age. Arrows indicate the pregnancy-associated lesion. (a) A sagittal T2-weighted MR image. (b) An axial T2-weighted MR image. (c) An enhanced axial MR image. (d) Gross pathology of the uterus and the pregnancy-related tissue. (e) An enlarged view of the implantation site.

A 40-year-old, gravida 5, para 2 patient with a history of two spontaneous miscarriages and one right tubal pregnancy treated with laparoscopic right salpingectomy consulted our hospital with a complaint of amenorrhea over the previous 7 weeks and 3 days. She had mild lower abdominal pain, and her vital signs were stable. The serum human chorionic gonadotropin (hCG) *β* level was 63,557 mIU/ml, which indicated pregnancy. Transvaginal ultrasonography (TV-USG) did not show a GS, but an eccentric, 38-mm heterogeneous mass was detected in the uterine interstitium. MRI was performed at 7 weeks and 5 days of pregnancy, and a heterogeneously enhanced hypervascular mass was identified on the right side of the uterine fundus (Figures 1(a)–1(c)). The mass measured 52 mm, which was larger than a GS at the corresponding estimated gestational age. An ectopic pregnancy was primarily suspected, but the possibility of GTD was also considered because of the atypical images and the size of the mass. She did not desire fertility preservation, and an abdominal hysterectomy was performed on the same day. The macroscopic contents of the mass included a villous component, an embryo, and a blood clot (Figures 1(d) and 1(e)). The crown-rump length (CRL) of the embryo was 10 mm, which corresponded to 7 weeks of pregnancy. Histopathologic examination showed normal villi. The final diagnosis was interstitial pregnancy. The postoperative course was uneventful, and she was discharged on postoperative day 7.

## 3. Case 2

A 38-year-old, gravida 3, para 2 patient with a history of 2 cesarean sections was referred to our department and hospitalized at 8 weeks and 3 days of pregnancy due to a suspected cesarean scar pregnancy. The hCG *β* level was 228,454 mIU/ml. TV-USG showed the GS with a live fetus on the cesarean scar of the uterus. The CRL was 19.3 mm. MRI findings also confirmed a cesarean scar pregnancy. The patient requested termination, but she wanted fertility preservation and decided to undergo medical treatment. Methotrexate and potassium chloride were locally injected under ultrasonic guidance at 9 weeks and 2 days of pregnancy.

**Figure 2:**
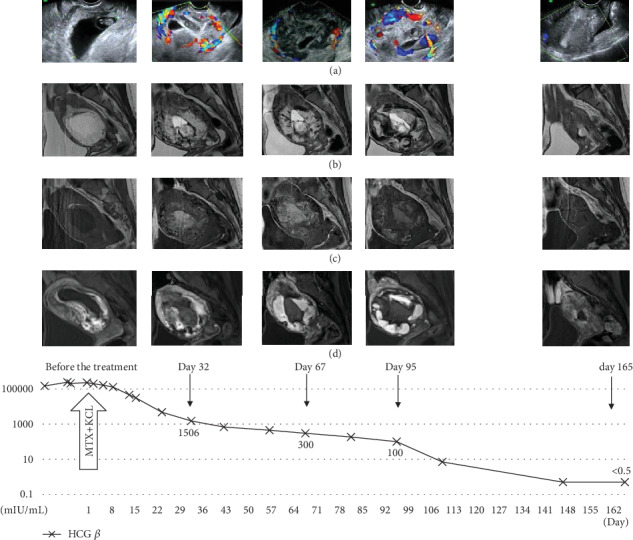
Serial images before and after medical treatment in case 2, a cesarean scar pregnancy, are shown. Transition of the serum hCG *β* level is shown by a graph of an exponential function. The lesion did not decrease in size and even increased despite a decrease in the serum hCG level. (a) TV-USG. (b) T2-weighted MR image. (c) T1-weighted MR image. (d) Enhanced MR image.

One month after treatment, the serum hCG *β* level had decreased to 1,506 mIU/ml. However, TV-USG revealed a hypervascular lesion in the uterus. Enhanced MRI showed a strongly enhanced hypervascular mass that was larger than that before the treatment (Figure 2). Sequential GTD was suspected because of the heterogeneous change and growth of the mass, but an invasive finding such as a fluffy appearance of tumor burden was absent on MRI; thus, the patient underwent careful follow-up. The lesion continued to grow gradually as shown on serial MRI on days 67 and 95; however, the size of the enhanced lesion did not change, and the enlarged part was considered to be a hematoma. The serum hCG *β* level continued to decrease. Menstruation restarted on day 120, necrotic tissue was extruded from the uterus on day 133, and the serum HCG *β* level became negative (<0.5 mIU/ml) on day 146. MRI was performed on day 165 and showed no residual disease in the uterus. There was no atypical bleeding or abdominal pain during the observation period. The final diagnosis was cesarean scar pregnancy.

## 4. Discussion

The differential diagnosis of ectopic pregnancy and GTD by imaging features is sometimes difficult. GTD is a group of rare tumors that can originate in any product of conception. Although GTD in ectopic pregnancy is quite rare, Tasha reported that GTD was found in approximately 18 of 100 ectopic pregnancies [3]. GTD is visualized as a heterogeneously hyperintense tumor on MRI [4]. In some cases of ectopic pregnancy, particularly interstitial, cervical, or cesarean scar pregnancy, the pregnancy-related tissue shows an atypical appearance that is described as a heterogeneous hypervascular mass on USG and MRI [5, 6]. When a hematoma exists around the mass, the size of the mass appears to be larger than that of the GS at the same gestational age. The embryo is obscured by the hematoma, which makes diagnosis difficult. The mass even shows spurious enlargement after medical treatment due to the formation of the hematoma [7]. This is probably because the muscle layer of the uterus stretches in an interstitial or cesarean scar pregnancy, which allows the formation of growing hematoma, whereas the fallopian tube tears easily in a tubal pregnancy. One of the differences between an ectopic pregnancy and GTD may be an invasive finding in the myometrium on MRI [4]. Actually, in our cases, the pregnancy-associated mass was heterogeneous and deceptively looked similar to ectopic GTD; however, the invasive findings were absent, and the radiologist correctly diagnosed them as ectopic pregnancies. In case 2, the ectopic lesion enlarged and showed heterogeneous change even after medical treatment, but the invasive finding was similarly absent. The reason why the lesion increased may be the expansion of hematoma in the residual villous tissue. As described, the lesion sometimes shows heterogeneous changes and grows in a nonmalignant ectopic pregnancy; thus, it is important to assess the myometrial invasion on MRI.

The management differs for an ectopic pregnancy vs. ectopic GTD. For ectopic GTD, the management is usually a combination of surgical removal of the conceptus and chemotherapy [8]. For an ectopic pregnancy, either surgical treatment or medical treatment is selected. Surgical treatment is conventionally chosen for an interstitial pregnancy and a cesarean scar pregnancy, but medical treatment has recently become more prevalent [9, 10]. When sufficient medical treatment is rendered in an ectopic pregnancy, the duration until the pregnancy-related tissue disappears varies from 1 to 6 months [10]. Some researchers report that the lesion vanishes before the serum hCG level becomes negative [11], while other researchers report that conversion to a negative serum hCG level occurs earlier [12]. In case 2, it took approximately 5 months until the remnant tissue was discharged. These findings indicate that even if the pregnancy-related tissue remains for an extended period or even shows growth after treatment in an ectopic pregnancy, it is possible to wait for a complete recovery with careful follow-up providing that the hCG level decreases and the patient's general status remains stable. To protect patients from overtreatment, additional studies are required to distinguish an ectopic pregnancy with confusing imaging features from GTD.

In conclusion, we reported two cases of ectopic pregnancy that posed difficulty in determining the differential diagnosis of GTD. MRI may lead to an accurate diagnosis of ectopic pregnancy and avoidance of unnecessary hysterectomy.

## References

[1] Panelli D. M., Phillips C. H., Brady P. C. (2015). Incidence, diagnosis and management of tubal and nontubal ectopic pregnancies: a review. *Fertility Research and Practice*.

[2] Tamai K., Koyama T., Togashi K. (2007). MR features of ectopic pregnancy. *European Radiology*.

[3] Tasha I., Kroi E., Karameta A., Shahinaj R., Manoku N. (2010). Prevalence of gestational trophoblastic disease in ectopic pregnancy. *Journal of prenatal medicine*.

[4] Dhanda S., Ramani S., Thakur M. (2014). Gestational trophoblastic disease: a multimodality imaging approach with impact on diagnosis and management. *Radiology Research and Practice*.

[5] Rheinboldt M., Ibrahim S. (2013). Atypical presentation of a large interstitial pregnancy. *Emergency Radiology*.

[6] Ackerman T. E., Levi C. S., Dashefsky S. M., Holt S. C., Lindsay D. J. (1993). Interstitial line: sonographic finding in interstitial (cornual) ectopic pregnancy. *Radiology*.

[7] Guzowski G., Sieroszewski P. (2014). Invasive ultrasound in the management of cervical ectopic pregnancy. *European Journal of Obstetrics, Gynecology, and Reproductive Biology*.

[8] Gillespie A. M., Lidbury E. A., Tidy J. A., Hancock B. W. (2004). The clinical presentation, treatment, and outcome of patients diagnosed with possible ectopic molar gestation. *International Journal of Gynecological Cancer*.

[9] Monteagudo A., Minior V. K., Stephenson C., Monda S., Timor-Tritsch I. E. (2005). Non-surgical management of live ectopic pregnancy with ultrasound-guided local injection: a case series. *Ultrasound in Obstetrics & Gynecology*.

[10] Pirjani R., Bayani L., Shirazi M. (2015). Successful local and systemic medical treatment of cesarean scar pregnancy and a subsequent term pregnancy after treatment: a case series. *Iranian Journal of Reproductive Medicine*.

[11] Dadhwal V., Deka D., Ghosh B., Mittal S. (2009). Successful management of live ectopic pregnancy with high beta-hCG titres by ultrasound-guided potassium chloride injection and systemic methotrexate. *Archives of Gynecology and Obstetrics*.

[12] Petousis S., Margioula-Siarkou C., Kalogiannidis I. (2015). Conservative management of cervical pregnancy with intramuscular administration of methotrexate and KCl injection: case report and review of the literature. *World Journal of Clinical Cases*.

